# Performance Portrait Method: Robust Design of Predictive Integral Controller

**DOI:** 10.3390/biomimetics10020074

**Published:** 2025-01-25

**Authors:** Mikulas Huba, Pavol Bistak, Jarmila Skrinarova, Damir Vrancic

**Affiliations:** 1 Institute of Automotive Mechatronics, Faculty of Electrical Engineering and Information Technology, Slovak University of Technology in Bratislava, Ilkovicova 3, 841 04 Bratislava, Slovakia; pavol.bistak@stuba.sk; 2 Department of Computer Science, Faculty of Natural Sciences, Matej Bel University, Tajovskeho 40, 974 01 Banska Bystrica, Slovakia; jarmila.skrinarova@umb.sk; 3 Department of Systems and Control, J. Stefan Institute, Jamova cesta 39, 1000 Ljubljana, Slovenia; damir.vrancic@ijs.si; 4 Faculty of Industrial Engineering Novo mesto, Segova ulica 112, 8000 Novo Mesto, Slovenia

**Keywords:** optimal control, robust control, PID control, predictive integral control, performance measures, performance portrait method

## Abstract

The performance portrait method (PPM) can be characterized as a systematized digitalized version of the trial and error method—probably the most popular and very often used method of engineering work. Its digitization required the expansion of performance measures used to evaluate the step responses of dynamic systems. Based on process modeling, PPM also contributed to the classification of models describing linear and non-linear dynamic processes so that they approximate their dynamics using the smallest possible number of numerical parameters. From most bio-inspired procedures of artificial intelligence and optimization used for the design of automatic controllers, PPM is distinguished by the possibility of repeated application of once generated performance portraits (PPs). These represent information about the process obtained by evaluating the performance of setpoint and disturbance step responses for all relevant values of the determining loop parameters organized into a grid. It can be supported by the implementation of parallel calculations with optimized decomposition in the high-performance computing (HPC) cloud. The wide applicability of PPM ranges from verification of analytically calculated optimal settings achieved by various approaches to controller design, to the analysis as well as optimal and robust setting of controllers for processes where other known control design methods fail. One such situation is illustrated by an example of predictive integrating (PrI) controller design for processes with a dominant time-delayed sensor dynamics, representing a counterpart of proportional-integrating (PI) controllers, the most frequently used solutions in practice. PrI controllers can be considered as a generalization of the disturbance–response feedback—the oldest known method for the design of dead-time compensators by Reswick. In applications with dominant dead-time and loop time constants located in the feedback (sensors), as those, e.g., met in magnetoencephalography (MEG), it makes it possible to significantly improve the control performance. PPM shows that, despite the absence of effective analytical control design methods for such situations, it is possible to obtain high-quality optimal solutions for processes that require working with uncertain models specified by interval parameters, while achieving invariance to changes in uncertain parameters.

## 1. Introduction

Automatic control is hidden behind most of the modern achievements in science and technology. And although these have changed and continue to change the life of the entire population to a considerable extent, the details of their functioning are not always sufficiently known. Despite their interdisciplinary nature, control problems are usually considered as part of the specific areas in which they are used, whether it is mathematics, computer science, electrical and electronic engineering, process control, motion control, telecommunications, power electronics and drives, smart and sustainable energy sources, agriculture, biology, medicine, etc. Of course, this distributed exploitation is then also reflected in the development of the unifying roof discipline focused on automatic control. As a result of the explosion of information in these particular areas, there can also be an insufficient understanding of the essence of the particular control problems. Existing restrictions affect human resources themselves, as well as the processes of research and dissemination of information. This can then result in the adoption of superficial, even if from certain aspects, seemingly fashionable, solutions. An illustrative example of these statements can be given from the field of proportional–integral–derivative (PID) controllers. These are considered the most commonly used technology for automatic control [1,2]. Despite the key role in automatic control, during the more than 8 decades from the appearance of the three-term Taylor Fulscope Controller, which was later (not quite correctly) labeled with the abbreviation PID, and thus gave the name to the whole discipline, no one noticed that it is actually a controller with a simplified disturbance observer (DOB) used for reconstruction and compensation for disturbances. And it was not even noticed by the pioneers using DOB-based control, who started using DOB to improve the performance achieved by means of PID [3,4,5,6] a few decades later. So, as commented for example by the authors of the IFAC survey [7], 90 years after the invention of PID, we still have nothing that compares with it. In other parts of the article, we will show that such a situation does not have to be unchanged in the future.

As stated in one of the basic textbooks in the field of PID control [8], many facts about PIDs were rediscovered just in connection with the shifts in technology. Even then, when it was necessary to repeat the solutions that had existed for years, but on a new technological basis [9], it turned out that several important facts about older solutions did not make it into the textbooks. The authors of successful commercial solutions simply did not feel the need to reveal the secret of their success, and a significant amount of essential knowledge was simply lost over time.

Returning to this period of already forgotten solutions proves to be useful also from the point of view of improving dead-time compensation in some types of control tasks. This will mainly involve the task of compensating the disruptive effects of the environment on the controlled processes by generating counter-oriented action interventions using negative feedback. Such applications have long been known, for example, in the field of acoustics. Under names such as active noise control (ANC), noise cancellation (NC), or active noise reduction (ANR), a method can be found to reduce unwanted sound by adding a second opposite sound. The concept was developed in the late 1930s, with commercial solutions becoming available in the late 1980s.

On a similar principle, magnetoencephalography (MEG) focused on measuring low-intensity magnetic fields arising as a result of brain or heart activity has been developed in recent decades [10,11,12]. MEG requires the elimination of the Earth’s magnetic field and of other sources of ambient magnetic fields that interfere with useful signals. This is possible in a certain frequency range with the use of magnetic shielding. In the area of low frequencies, where the shielding is less effective, this must be supplemented by the generation of an oppositely acting magnetic field created by the current passing through the system of suitably placed coils. For the compensation to be successful, the opposing magnetic fields must be generated quickly enough. The main source of delay seems to be the sensors needed to detect the surrounding magnetic fields. Among them, optically pumped magnetometers (OPMs) [13,14] are gaining ground. Their dynamics can be approximated by the transport delay (to which several smaller time constants of other members of the control loop also contribute) and one dominant time constant. Previous contributions to the design of compensation loops are based on the use of PID [15] and PI controllers [16,17,18]. As for the identification of the sensor used and the design of the controllers, these articles do not provide any detailed information. One of the first exceptions in this direction is the recent work [19]. In applications using the QuSpin zero-field magnetometer (QZFM) to diagnose cardiovascular diseases, a PI controller design is used based on the approximation of sensor dynamics with a first-order time constant. However, during the experimental evaluation, its lower effectiveness was noted and the addition of reconstruction and compensation of disturbances using active disturbance rejection control (ADRC) was suggested. In this context, however, the question arises as to whether an approximation of the entire loop containing several other smaller delays, including actuator delays and delays arising from computer circuitry, with a single time constant is appropriate. Necessary arguments that would show its justification compared to the intuitively expected approximation using pure dead-time or a combination of first-order time delayed (FOTD) dynamics are missing. Suspicions of incorrect sensor dynamics modeling are also raised by another recent work [20].

In it, the OPM element is approximated by the FOTD model with a time constant $T_{a}\approx 1$ ms and dead-time $T_{d}\approx 3.2$ ms. This represents a significant shift compared to the previous interpretation. Because the pure delay element is inherently infinite-dimensional, the authors avoid direct analysis of the circuit by using the Padé approximation of the dead-time element. Next, to design the controller, they use generalized active disturbance rejection control (GADRC). When using the Padé approximation of the third order, this leads to the use of controllers of the fifth order. Today, in the MEG field, dissatisfaction with PI and PID control is demonstrated using ADRC. However, ADRC is based on DOB design using state-space approaches that were not primarily developed for systems with dead-time [21], and the use of Padé approximation unnecessarily complicates them. Therefore, it is appropriate to look for more direct and simple approaches.

Let us add that the emerging situation with the dominant dynamics located in the feedback was already dealt with in advance [22,23,24,25]. The inspiration for their writing was the widespread use of PI and PID controllers even in situations where they do not represent a meaningful solution. And they provided similar conclusions from the point of view of using Smith’s predictor, which was also originally designed for other situations [26,27,28]. However, the aforementioned works focused on the analysis and design of setpoint responses, since for MEG, the dynamics of disturbance responses is dominantly important. To overcome the problems resulting from the presence of a pure delay representing an infinite-dimensional element, the analysis and synthesis of the circuit using the performance portrait method (PPM) can be applied.

The rest of this paper is structured as follows. Section 2 deals with the optimization of the controller design using more advanced performance measures based on the modifications of the monotonicity criterion with respect to different dynamic control classes. Section 3 analyzes the two basic options for controlling the system with a pure dead-time element located in the circuit’s feedback and shows that the quality of circuits using I, PI, or PID controllers can be significantly improved with the use of a controller inspired by Reswick’s disturbance–response feedback concept. Section 4 provides an introduction to the predictive I-controller design by the performance portrait method. A more detailed analysis and synthesis of Reswick’s system extended by the use of low-pass filters of various orders using PPM is treated in Section 5. The discussion of the results achieved and the possibility of their extension to the approximation of the sensor dynamics also with the consideration of other time constants is given in Section 6. The summary of the work and the possibility of further extension of the achieved results are given in Section 7.

## 2. Controller Optimization Considering Dynamic Classes of Control

Optimization in the field of PID controller design is mainly realized using the integral of absolute error (IAE) with the addition of restrictions based on the peaks of sensitivity functions [1,8,29,30]. The integral of the absolute error is defined for the setpoint and disturbance steps as

(1) $$IAE=\int_{0}^{\infty }\left|e(t)\right|dt\;\;;\;\;\;e=w-y\;\;$$

In controller design, it is recommended to consider both the setpoint and disturbance responses. For the setpoint steps with $w_{s}=1$, input disturbance $d_{is}=0$, and $y=y_{s}$, it will be denoted as $IAE_{s}$. For the input disturbance steps with $w_{d}=0,d_{id}=1$, $y=y_{d}$, it will be denoted as $IAE_{d}$.

Among other performance measures, monotonicity index [29,30] and total variation $TV\approx \sum_{i}\left(\left|y_{i+1}-y_{i}\right|\right)$ of control signal [31] can be mentioned. Both these performance measures are based on summing absolute values of the smallest increments of considered signals. The monotonicity index, interesting for the evaluation of setpoint steps at the process output, can be achieved by relating the sum of all output increments to the sum of their absolute values. Its value then takes figures from 1 (for monotonic responses) to zero.

Another formulation of monotonicity of setpoint responses $y_{s}$ can be calculated with a given sampling period $T_{s}$ from TV as

(2) $$\begin{array}{c} TV_{0}\left(y_{s}\right)\approx \sum_{i}\left(\left|y_{i+1}-y_{i}\right|\right)-\left|y_{\infty }-y_{0}\right| \end{array}$$

For a fully monotonic response, $TV_{0}\left(y_{s}\right)=0$, else $TV_{0}\left(y_{s}\right)>0$. The advantage of this expression of monotonicity is the possibility of evaluating deviations from the ideal shape of signals consisting of several monotonic segments. For example, an ideal disturbance response has to consist of two monotonic intervals forming a one-pulse (1P) shape. The first interval with a monotonic increase in output deviation after disturbance step should ideally be followed by a monotonic return to the required setpoint value. These two monotonic intervals are separated by an extreme point $y_{m}\notin \left(y_{0},y_{\infty }\right)$ representing a turning point. By applying performance evaluation according to (2) on these two intervals then yields a modified TV measure

(3) $$TV_{1}\left(y_{d}\right)=\sum_{i}\left|y_{i+1}-y_{i}\right|-\left|2y_{m}-y_{\infty }-y_{0}\right|$$

The possibility of formulating performance measures to express deviations from ideal shapes consisting of m≥0 monotonic intervals at the input of the process makes it possible to significantly enrich the spectrum of performance measures useful for process optimization. For their interpretation, we will first introduce the concept of dynamic control classes.

As an alternative to finding the minimum IAE values constrained by the allowable excessive controller effort, it can be considered minimization of a combined cost function *J* defined as

(4) $$J=IAE*TV_{0}\left(u\right)$$

### Dynamic Classes of Control

It is worth noting that the design of PID controllers, which are still dominant in practice, is mainly oriented towards the design of stable systems [29,30,32]. And because, in the case of stable processes, there are several possibilities of specifying the target dynamics, without precisely naming the reasons for this “degrees of freedom”, the design of PID controllers is associated with the inflation of solutions [32].

In connection with the definition of the ideal shapes of target responses at the process input and output, another neglected concept of dynamic classes of control should be mentioned, which, despite having a key role in the optimal controller design [33,34] and being published in a modified version at leading world conferences and in top journals, has not yet become widespread in PID control [35,36].

In order to be able to introduce a system into the optimal design of controllers, it is necessary to define the possible basic classes of behavior of dynamic processes by defining the expected forms of transients at their input and output corresponding to step changes of input signal. The dynamic classes will be derived by generalizing the concept of monotonicity denoting the simplest possible form of changes of a time-varying signal. By considering a signal with several monotonic segments, the concept of mP-function can be created.

**Definition** **1** (mP function u(t)). *Let u(t) be a function corresponding to the closed-loop control signal of a stable jth-order linear time-delayed system that is:*

- *Continuous for t∈(0,T),T→∞;*
- *With possible discontinuity at $t=0^{+}$;*
- *With initial value $u_{0}=u\left(0^{-}\right)$ and final steady-state value $u_{T}=u\left(T\right)$.*

*Let in a step response u(t) which can be found for 0<t<T m extremes (0≤m≤j) lying alternately over and below (or vice versa) the level $u_{T}$ and fulfilling (with the denotation $u_{i}=u\left(t_{i}\right);\;i=1,2,\ldots ,m\;\;\mathrm{for}\;\;0<t_{1}<\ldots <t_{m}$) conditions*

(5) $$\begin{array}{c} \left(u_{i}-u_{T}\right)\;\left(u_{i+1}-u_{T}\right)<0;\;i=1,2,...,m-1 \end{array}$$

*Then, the function u(t), which is monotonic on each of the m+1 intervals not containing one of the above extreme points $u_{i},i=1,2,...,m$, is called m-Pulse (mP) function.*

*In case of discontinuity at t=0, the first extreme point can also be moved to the origin $t=0^{+}$ (i.e., $u_{1}=u\left(0^{+}\right)$), thus shrinking the first monotonic interval between $u_{0}$ and $u_{1}$ with $t\in (0^{-},0^{+})$ to zero.*

**Definition** **2** (Dynamical classes of control). *For a non-negative integer N, dynamical class of control DCN [35] denotes control with all setpoint step responses given by NP input–0P output pairs u(t),y(t). In other words, it denotes setpoint step responses with the plant input u(t),t∈(0,∞) consisting of N+1 alternately monotonically increasing and decreasing (or vice versa) segments, which are associated with the monotonic plant output y(t),t∈(0,∞).*

*In the case of the disturbance step responses, it denotes NP input–1P output pairs u(t),y(t) with the plant input u(t),t∈(0,∞) consisting of N+1 monotonic segments, which are associated with the plant output y(t),t∈(0,∞) consisting of two monotonic response segments.*

The research community approached the introduction of the concept of dynamic classes of control already in the first “fashionable” wave of research after the Second World War. Because it was inspired mainly by military applications, in which the basic goal of the design was to overtake the opponent, it focused mainly on optimal control, in which time-optimal control dominated. Of the practically oriented results, Feldbaum’s principle of *n*-optimal intervals of relay time-optimal control can be mentioned here [33]. In the time-optimal control of *n*th-order systems with a full relative degree, it brought the first systematic requirement to terminate the process in *n* (rectangular) pulses (control intervals) [33]. Although Felbaum’s results have been later overshadowed by more broadly formulated modifications resulting from the maximum/minimum principle [34], some differences appeared just for the case of systems with complex poles and only for a large distance between the initial and final states. When moving from one steady state to another, the differences do not apply.

In the case of systems with real poles, the conclusions of Feldbaum’s theorem could be formulated in a simplified form:

**Theorem** **1** (Feldbaum Theorem). *For linear systems with j real poles and full relative degree r=j, after a step change of the reference setpoint or the disturbance, the number of control signal intervals with a limit control signal value required to achieve the neighborhood of the desired state, with piece-wise alternating control signal limits, is equal to j.*

In engineering practice, the relay time-optimal transients appear only exceptionally. Greater emphasis is placed on smooth continuous changes of the control signal and well-damped steady states. In the time-optimal control developed for military applications (as missile control, fire control, etc.), the steady states were frequently not considered. Therefore, due to different priorities, in the literature focused on PID controllers, Feldbaum’s theorem has disappeared [9,37]. In addition, when controlling stable systems, the effectively observed number of pulses may be less than the order of the system under consideration. In this respect, it is more important that, with sufficiently smooth and slow processes, it is possible to formulate the following Lemma (see [36]):

**Lemma** **1.** *In each Bounded–Input–Bounded–Output (BIBO) stable system, after a step change of the reference setpoint or disturbance, a neighborhood of the desired state may be achieved with monotonic setpoint step responses of the controller output (plant input) u(t).*

However, with some exceptions such as those aforementioned [9,37], which continued to use Feldbaum’s theorem on optimal control also for smoother control responses, this feature of relay time-optimal controllers dealing with the number of optimal control pulses disappeared from the PID control over time. However, recently, the ever-increasing demands placed on the performance of PID control have been fulfilled by using controllers with higher-order derivatives [38]. This leads to the fact that, when evaluating its dynamics of transients, it will again be necessary to pay attention to control responses possibly obtaining a higher number of pulses.

## 3. Illustrative Example: Controllers for Process Models with Pure Dead-Time and Dominant Dynamics in the Feedback

Control theory demonstrates that an appropriate model of the environment is a key component of any successful control system [39]. The simplest models of stable dynamical systems can be formulated in the form of a pure dead-time. To follow a stepwise constant reference signal w(t), for a stable uncertain process with a dominant dead-time $T_{d}$, process input u(t), and process output y(t), such a model can be formulated in Laplace transform as

(6) $$\begin{array}{c} S\left(s\right)=\frac{Y(s)}{U(s)}=Ke^{-T_{d}s}\\ K\in \left[K_{min},K_{max}\right]\;;\;T_{d}\in \left[T_{min},T_{max}\right] \end{array}$$

Its two main parameters—the gain *K* and the dead-time $T_{d}$, can either be variable in time, dependent on the process inputs and states, or not precisely known.

### 3.1. Short Overview of Existing Solutions

Since a large number of processes from practice can be approximated using the pure-dead-time model, attention has been paid to the control design for such models from early control origins. The solutions for such a process were also interesting because several actuators (electrical and hydraulic motors) can be described as integrators. The I-controller has as an input the control error e(t)=w(t)−y(y), where w(t) represents a reference setpoint value. When unified with an actuator with u(t) at its output, it can be described as

(7) $$C\left(s\right)=\frac{U(s)}{E(s)}=\frac{1}{KT_{f}s};\;E\left(s\right)=W\left(s\right)-Y\left(s\right)$$

with the time constant $T_{f}$ specified by the double real dominant pole method as

(8) $$T_{f}=e^{1}T_{d}\approx 2.71T_{d}$$

This was already proposed in one of the first textbooks on automatic control by Oldenbourg and Sartorius [40], representing also one of the first uses of MRDP design. Value (8) has a special role also in the robust design of this controller which is treated in [23].

The first known work in the field of dead-time systems, which presented a controller including a dead-time model, was presented by J. B. Reswick [41]. The new control concept was introduced as a disturbance–response feedback. And although some later works [42,43,44] focused on its further elaboration, over time it fell into oblivion and was overshadowed by another pioneering work from this area by O.J.M. Smith [26]. However, Smith’s Predictor considered the control of a stable time-delayed first-order process in the feed-forward path, which is already a task from DC1. The control of pure-dead-time compensating systems is primarily a task from DC0. The reasons for forgetting Reswick’s work, which was not based on the dominant industrial control hardware, can also be connected with the need to work in the new control concept with the dead-time model, which was difficult to implement within the scope of available analogue controllers. However, as we will show later, the weakness of Reswick’s concept was also the lack of robustness of the proposed solution.

Skogestad, in the scope of SIMC (SIMple Control) design [31], proposed a pure dead-time system integral (I) controller by using results obtained by controlling the delayed integral process by the P-controller. For the given situation, he recommends the I-controller

(9) $$C\left(s\right)=\frac{1}{KT_{i}s};\;\;T_{i}=2T_{d}$$

with settings guaranteeing the maximal sensitivity value $M_{s}=1.59$. The I-controller, however, could not be configured with the most common kits for PID control. Therefore, Skogestad also compares the proposed solution (9) with seven other controllers, of which he recommends the PI controller according to Astrom/Schei

(10) $$C\left(s\right)=\frac{0.2}{K}\left(1+\frac{1}{T_{i}s}\right);\;\;T_{i}=0.3180T_{d}$$

with the parameters set by maximizing the I-action gain and guaranteeing $M_{s}=1.6$. He does not recommend the use of PID controllers with derivative action. However, since the recommendation to use just the I-action is outside the scope of the usual offer of industrial PID controllers, which, as a rule, did not make it possible to configure zero values of P and D action, working just with the I-component was not very popular.

In the next section, we will therefore review the approaches resulting from the dominance of PID control and try to compare them with the procedures inspired by the original proposal by Reswick.

### 3.2. Approximation of Filtered Dead-Time Inversion

The first intuitive solution could be based on replacing inversion of the dead-time model $e^{T_{m}s}$ with the highest possible number of terms *m* of the Taylor expansion

(11) $$e^{T_{m}s}\approx 1+\frac{1}{1!}T_{m}s+\frac{1}{2!}{\left(T_{m}s\right)}^{2}+\ldots +\frac{1}{m!}{\left(T_{m}s\right)}^{m};\;m=0,1,2,\ldots$$

**Figure 1:**
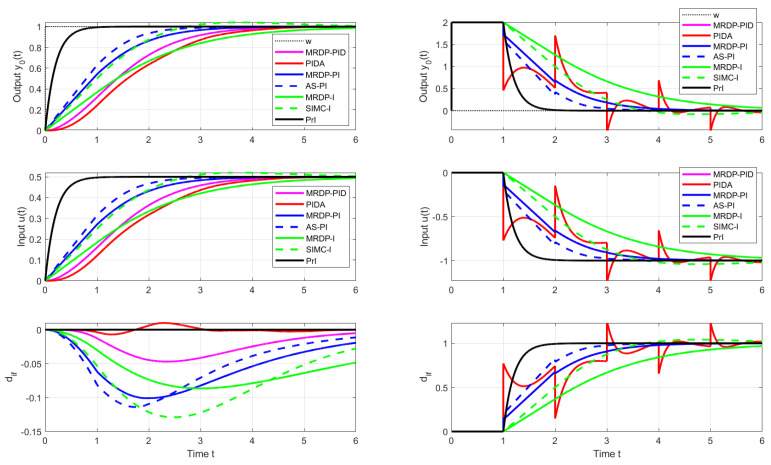
Setpoint (**left**) and input disturbance (**right**) step responses of the system (6) achieved with I, PI, PID, PIDA, and PrI controllers.

With such a procedure, however, we will encounter limitations resulting from the use of higher-order filters necessary to implement the higher-order output derivatives. Figure 1 shows the responses of the setpoint and disturbance step responses of the circuit with approximation of $e^{sT_{d}}$ specified by m=n=4 for $T_{d}=T_{m}=1$, $K=K_{m}=2$ and $T_{f}=0.6$. It is obvious that the response to the disturbance step (red curve) contains a significantly oscillating component. Such a system can be to an equivalent circuit with a controller

(12) $$C\left(s\right)=\frac{T_{1}}{3KT_{f}}\left(1+\frac{1}{T_{1}s}+\frac{T_{2}s}{T_{1}}+\frac{T_{3}s}{T_{1}}\right)\frac{1}{1+sT_{f}+s^{2}T_{f}^{2}/3}$$

that can be denoted as a proportional–integral–derivative–acceleration (PIDA) controller. This should yet be completed by a pre-filter

(13) $$F_{p}\left(s\right)=\frac{1}{1+T_{1}s+T_{2}s^{2}+T_{3}s^{3}}.$$

The oscillatory component is introduced by the filter of the equivalent PIDA controller (12) having the polynomial $1+sT_{f}+s^{2}T_{f}^{2}/3$, which contributes to the pole s=0 by complex poles

(14) $$s_{1,2}=\frac{3}{2T_{f}}\left(-1\pm j\sqrt{\frac{1}{3}}\right)$$

In the case of setpoint steps, the control signal grows only slowly, so the contributions of these complex fields are not excited. Their weight coefficients in the overall solution of the system are small. In the case of disturbance responses, however, sharp changes in the control signal lead to a stronger excitation of complex components and to an increase in their contribution to the overall solution, which increases with the reduction in $T_{f}$. Due to this, the responses after the disturbance steps are sufficiently smooth only for relatively large $T_{f}$. At $T_{f}=T_{d}/2$, the fast oscillatory components of the solution already cause instability.

As m=n increases, the influence of complex components in the reconstructed disturbance increases. For m=n=4, the difference ${\left(1+sT_{f}\right)}^{4}-1$ will contribute with poles $0,-2/T_{f}$ and $\left(-1\pm j\right)/T_{f}$. For m=n=5, ${\left(1+sT_{f}\right)}^{5}-1$ contributes with poles $0,\left(-0.69\pm j0.95\right)/T_{f}$ and $\left(-1.81\pm j\pm j0.59\right)/T_{f}$.

With regard to the given problem caused by the positive feedback from the controller output, it is appropriate to limit the maximum order of the filter $Q_{n}\left(s\right)$ to the value n=2. At the same time, it also limits the maximum value of *m* in (11) and thus it will not be possible to increase the degree of the used controller as when using generalized PID and automatic-reset controllers in [38]. The appearance of possible fast oscillatory components due to coarser approximation can be avoided by designing the loop using the multiple real dominant pole (MRDP) method, which has been used for controller design since one of the first automatic control textbooks [38,40,45].

For m=n=2, the approximative design yields parameters and equivalent PID controller

(15) $$\begin{array}{c} T_{f}=0.92298;\;T_{1}=0.7117581999;\;T_{2}=0.1154823911\;\\ C\left(s\right)=\frac{T_{1}}{K2T_{f}}\left(1+\frac{1}{T_{1}s}+\frac{T_{2}s}{T_{1}}\right)\frac{1}{1+sT_{f}/2} \end{array}$$

For m=n=1 and $T_{d}=1$ with a quasi-polynomial $A\left(s\right)=T_{f}e^{s}+1+T_{1}s$ MRDP design yields

(16) $$T_{1}=1/4;\;Tf=e^{2}/4=1.8473,$$

which corresponds to a PI controller

(17) $$C\left(s\right)=\frac{T_{1}}{KT_{f}}\left(1+\frac{1}{T_{1}s}\right)$$

The use of these controllers must be combined with the pre-filter (13), in which $T_{3}=0$ or $T_{2}=0$ is considered.

It is interesting that the MRDP-PI (17) and the MRDP-PID (15) controllers give the same disturbance response and the equivalent PID controller uses a series filter with a fixed time constant $T_{f}/2$.

For comparison, the SIMC I-controller (9) would correspond to m=0 and n=1 with the value $T_{f}=2$. The PI controller (10) denoted as Astrom/Schei corresponds to parameters m=n=1 and $T_{f}=1.5898,T_{1}=0.3180$.

In Figure 1, SIMC transients provide a bit faster than the controllers corresponding to MRDP design, but connect with a certain overshoot. In the nominal case, however, they do not reach the speed of responses inspired by Reswick’s disturbance–response feedback with the simplest DOB filter of the first order. In the case of controllers based on the dead-time element inversion approximation, experiments and derivation of equivalent controllers show that increasing the approximation order, which also requires the use of low-pass filters of a higher order, does not improve the dynamics even in the nominal case. In the case of DOBs with a dead-time element included in the positive feedback loop from the controller output, simply examining the properties of this loop is not so simple. The properties of the corresponding circuit depending on the used filter $Q_{n}\left(s\right)$ and the uncertainty of the circuit will be further examined using the performance portrait method (PPM).

### 3.3. Reconstruction and Compensation of a Delayed Input Disturbance

For a static process with gain *K*, the required output *w* can be set most easily using static feed-forward $1/K_{m}$. In order to obtain a smooth output response to setpoint steps, it requires the addition of a low-pass pre-filter. However, to compensate for an inaccurate model ($K_{m}\neq K$) and to compensate for the impact of external influences characterized by the input (mostly unmeasurable) disturbance $d_{i}=const$, the circuit must be supplemented with reconstruction and compensation of $d_{i}$.

**Figure 2:**
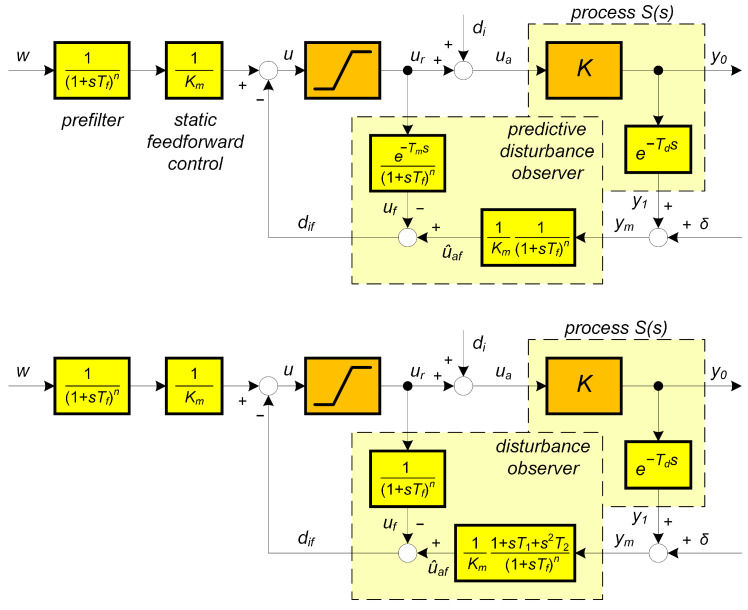
Two possible implementations of static feed-forward with disturbance reconstruction and compensation for the pure-dead-time model of the dynamical class 0 inspired by Reswick’s disturbance–response feedback concept; δ—measurement noise.

The input disturbance $d_{i}$ can be reconstructed as the difference between the actual input to the process $u_{a}$ and the output from the controller *u* limited to the value of $u_{r}$ by respecting the admissible control signal constraints. There are two ways to respect the transport delay $e^{-T_{d}s}$ - with the dead-time model $e^{-T_{m}s}$ (Figure 2 above), or by an approximative inversion of the dead-time model (shown for $e^{T_{m}s}\approx 1+T_{1}s+T_{2}s^{2}$ in Figure 2 below).

The value of $u_{a}$ can be reconstructed from the time-delayed output $y_{1}\left(t\right)=y_{0}\left(t-T_{d}\right)$ only with the use of the inverse gain of the process $1/K_{m}$, which gives the actual input estimate

(18) $${\hat{u}}_{a}\left(t-T_{d}\right)=y_{1}\left(t\right)/K_{m}=y_{0}\left(t-T_{d}\right)/K_{m}$$

The delay of the reconstructed actual input of the process must be respected by comparison with the delayed output of the controller according to the relationship

(19) $$d_{i}\left(t-T_{d}\right)={\hat{u}}_{a}\left(t-T_{d}\right)-u\left(t-T_{d}\right)$$

This leads to the correction of the reconstructed disturbance according to Figure 2 above inspired by Reswick’s disturbance–response feedback concept. However, unlike the starting work, the reconstruction relations are supplemented by a low-pass filter of the *n*th order

(20) $$\begin{array}{c} Q_{n}\left(s\right)=1/{\left(T_{n}s+1\right)}^{n}=1/M_{n}\left(s\right);\;n=1,2,... \end{array}$$

Then, the “predictive disturbance observer” (PDO) is used to obtain the filtered value of the disturbance reconstruction $d_{if}$ taking into account the estimate $T_{m}$ of the process delay $T_{d}$. By considering $Q_{n}\left(s\right)$ also as a pre-filter, the setpoint-to-output closed loop transfer function of the corresponding controller between the output $y_{1}$ and the setpoint *w*, or input disturbance $d_{i}$, can be derived as

(21) $$\begin{array}{c} F_{w}\left(s\right)=\frac{Y_{1}\left(s\right)}{W(s)}=\frac{Ke^{-T_{d}s}}{K_{m}\left({\left(T_{n}s+1\right)}^{n}-e^{-T_{m}s}\right)+Ke^{-T_{d}s}}\\ F_{i}\left(s\right)=\frac{Y_{1}\left(s\right)}{D_{i}\left(s\right)}=\frac{KK_{m}e^{-T_{d}s}\left({\left(T_{n}s+1\right)}^{n}-e^{-T_{m}s}\right)}{K_{m}\left({\left(T_{n}s+1\right)}^{n}-e^{-T_{m}s}\right)+Ke^{-T_{d}s}} \end{array}$$

The controller will be denoted as “predictive I-controller” (PrI). The belonging of its transients to DC0 can be stressed by the corresponding index “0” as PrI_0_ [22,24]. However, since the transients achieved using I and PrI controllers always belong to DC0, the index can also be omitted. Alternatively, the use of the index can be saved preferably for marking the degree of the used filter.

In the nominal case with $T_{d}=T_{m}$, $K=K_{m}$, integrals of error (IE) calculated by means of Laplace transform give values

(22) $$IAE_{s}=T_{d}+nT_{f}=T_{d}+T_{e};\;IAE_{d}=K\left(T_{d}+nT_{f}\right)=K\left(T_{d}+T_{e}\right);\;T_{e}=nT_{f}$$

When the control error does not change its sign, IAE=IE. For $T_{d}\neq T_{m}$ and $K\neq K_{m}$, integrals of error (IE) yield

(23) $$IAE_{s}=\left(T_{m}+T_{e}\right)K_{m}/K;\;IAE_{d}=K_{m}\left(T_{m}+T_{e}\right).$$

Here, $T_{e}=nT_{f}$ can be denoted as an equivalent delay of the filter. Because $T_{e}$ slows down transient responses, it would be appropriate to choose it as small as possible. However, with regard to measurement noise attenuation and also with regard to the robustness of the circuit, it will be necessary to find the smallest possible value of $T_{e}$ that meets these additional restrictions.

Nominal setpoint and disturbance step responses of the system (6) with $T_{d}=T_{m}=1$, $K=K_{m}=2$ with n=1 and $T_{f}=0.2$ are in Figure 1 given by black curves.

With respect to $F_{w1}\left(0\right)=1$, it is possible to claim that, in a steady state (if achieved), the output should track the given (constant) setpoint *w* also for a plant-model parameter mismatch with $K_{m}\neq K$ and $T_{m}\neq T_{d}$. From $F_{di1}\left(0\right)=0$, it follows that this scheme guarantees the rejection of piece-wise constant disturbances $d_{i}$ also for $K_{m}\neq K$ and $T_{m}\neq T_{d}$. Both these properties hold also for the output $y_{0}$ measured at the dead-time input, when

(24) $$F_{s0}\left(s\right)=F_{w1}\left(s\right)e^{T_{d}s}\;;\;\;F_{i0}\left(s\right)=F_{i1}\left(s\right)e^{T_{d}s}$$

**Remark** **1** (Specifics of higher-order approximations of dead-time inversion). *Approximation of the dead-time inversion according to Taylor (11) obviously does not represent a solution suitable for further development. The parameters of the controllers calculated using MRDP do not correspond to (11) and have to be linked to the parameters of the used filter. Increasing the approximation degree m does not lead to an improvement in setpoint and disturbance responses. Unlike the generalized design of PID and automatic reset controllers in [38], however, in dead-time inversion (11) the MRDP design is not suitable for higher-order approximations with n≥m>2. The comparison in Figure 1 shows, from the point of view of performance improvement, a much better potential of the solutions based on Figure 2. However, it will again be important to examine more deeply the influence of the choice of the filter $Q_{n}\left(s\right)$ also in the context of aspects of robustness and noise attenuation.*

**Figure 3:**
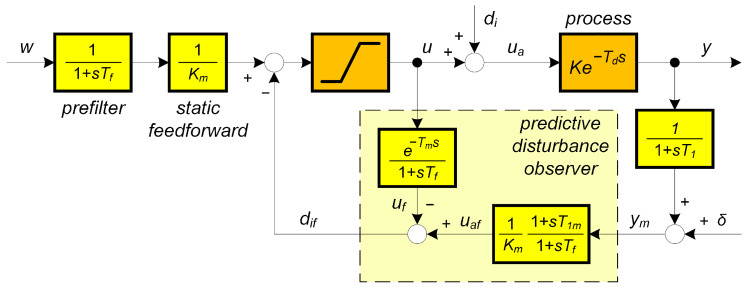
Predictive PI (PrPI) controller with compensation of a dead-time $T_{d}$ based on its estimate $T_{m}$ and of single sensor time constant $T_{1}$ based on its estimate $T_{1m}$; $K_{m}$ is the estimate of the process gain *K*; δ= measurement noise.

**Remark** **2** (Modifications of PrI controller). *In addition to the already mentioned reasons for using higher-order filters (measurement noise attenuation, closed-loop robustness), another important aspect is the possibility of compensating some additional time constants of sensor dynamics. Compensation of one time constant can be realized already with a first-order filter (see Figure 3). However, such a solution is frequently unsuitable from the point of view of noise attenuation. An equivalent solution labelled as predictive PI-controller (PIP, or PPI) with three adjustable parameters was proposed by Hägglund [46,47]. The PPI controller has already been available as an industrial solution.*

### 3.4. PPI Controller by Hägglund

Hägglund [46,47] designed his PPI controller at the time of PID control dominance, which influenced the way of its presentation, which should be as close as possible to the solutions in use. At the same time, he tried to reduce the number of optional system parameters as much as possible. Thus, the simplified controller was characterized by the equation

(25) $$u\left(t\right)=K_{c}\left(1+\frac{1}{T_{i}s}\right)e\left(t\right)-\frac{1}{T_{i}p}\left(u\left(t\right)-u\left(t-T_{d}\right)\right)$$

This makes it possible to derive the controller transfer function as

(26) $$C\left(s\right)=\frac{U(s)}{E(s)}=K_{c}\frac{1+T_{i}s}{1+T_{i}s-e^{-T_{d}s}}$$

When describing the process with parameters $K,T_{d}$, and $T_{1}$, Hägglund recommended the setting

(27) $$K_{c}=\frac{1}{K};\;T_{i}=T_{1}$$

He also proposed a controller tuning approach based on approximating the process step response by a gain, dead-time, and a time constant.

Controller transfer function corresponding to Figure 3 with $K_{m}=K$ and $T_{1m}=T_{1}$ is

(28) $$C\left(s\right)=\frac{U(s)}{E(s)}=\frac{1}{K}\frac{1+T_{1}s}{1+T_{f}s-e^{-T_{d}s}}$$

Comparison with (26), (27) shows a match for $T_{i}=T_{f}=T_{1}$ in the denominator of C(s) (28).

## 4. Performance Portrait Based Interpretation of PrI Controller

The formulation of PPM was made possible by the design of performance measures for evaluating the basic features of transient responses in [22]. Several of them were defined on the basis of monotonicity and used in combination with the definition of dynamic classes of control in [35].

Broad use of the PP method is ensured by enabling to generate the PP corresponding to a particular control loop just once and to use it repeatedly for controller tuning corresponding to specific plant parameters and performance constraints [48].

Intuitively, one could expect optimal behavior of the PrI controller for $K_{m}=K,T_{m}=T_{d}$. However, how to optimally choose $K_{m}$, $T_{m}$, and the parameters of $Q_{n}\left(s\right)$ in the case of a parameter mismatch? This information can be obtained by calculating a performance portrait (PP) in a sufficient number of grid points covering the relevant ranges of process and controller parameter changes. The repeated use of PP can be ensured by calculating it for standardized circuit parameters. For a given process (6) and controller according to Figure 1 above with some chosen $Q_{n}\left(s\right)$, it will be mapped for the setpoint and disturbance step responses by using a 3D coordinate system $\left(\kappa ,\tau_{f},\tau_{d}\right)$ with normalized (dimensionless) variables

(29) $$\begin{array}{c} \kappa =K/K_{m}\;;\;\;\tau_{f}=T_{f}/T_{m}\;;\;\;\tau_{d}=T_{d}/T_{m}\;;\;\;\tau_{p}=T_{p}/T_{m}\;;\;\;p=T_{m}s \end{array}$$

For the considered process model (6), such a task was solved for the first time in [22], but only considering PP created by evaluating and saving the setpoint responses property. With regard to the application in MEG, we will now expand the task to include the evaluation of input disturbance responses.

**Figure 4:**
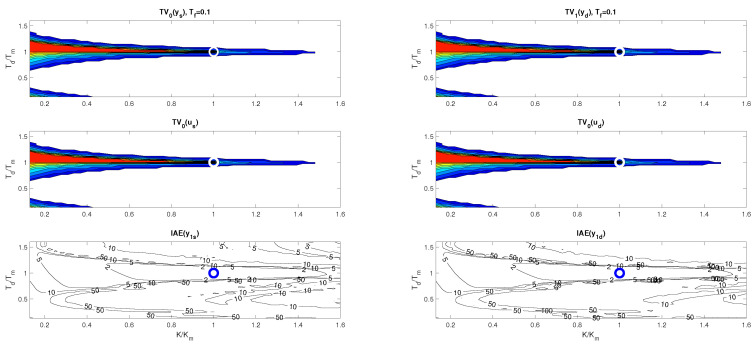
Cross-section of 3D PP for the value $\tau_{f}=0.1$ visualized according to (31), (32) for the vector of levels $v_{\epsilon }=\left[0.001\;0.002;0.005\;0.01\;0.02\;0.05\;0.1\;0.2\;0.5\;1\right]$ with the colors ranging from red to dark blue; the nominal setting $K_{m}=K$ and $T_{m}=T_{d}$ marked with a circle.

**Figure 5:**
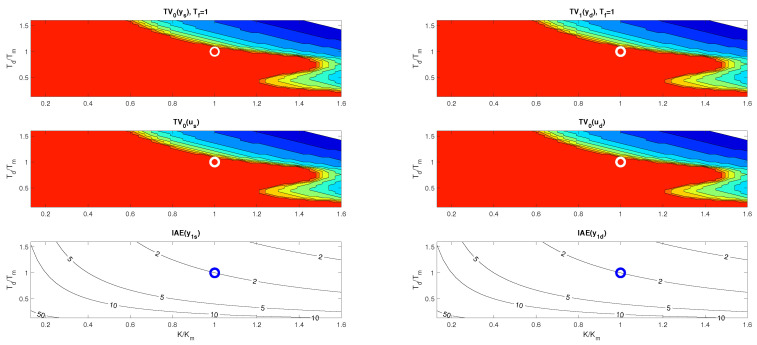
Cross-section of 3D PP for the value $\tau_{f}=1$ visualized according to (31), (32) for the vector of levels $v_{\epsilon }=\left[0.001\;0.002;0.005\;0.01\;0.02\;0.05\;0.1\;0.2\;0.5\;1\right]$ with the colors ranging from red to dark blue; the nominal setting $K_{m}=K$ and $T_{m}=T_{d}$ marked with a circle.

Figure 4 and Figure 5 show two boundary cross-sections of PP generated for n=1 with $K_{m}=1$ and $T_{m}=T_{p}=1$ in 50 × 50 × 10 points of the coordinates grid specified by

(30) $$\begin{array}{c} \kappa \in \left[0.13,1.6\right];\;\Delta \kappa =0.03;\\ \tau_{d}\in \left[0.13,1.6\right];\;\Delta \tau_{d}=0.03;\\ \tau_{f}=\tau_{e}\in \left[0.1,1\right];\;\Delta \tau_{f}=0.1. \end{array}$$

PPs in Figure 4 and Figure 5 confirm that there are no major differences between setpoint and disturbance responses as for higher-order controllers based on Taylor’s dead-time approximation in Figure 1. Nevertheless, with regard to applications in MEG control, the interpretation of the obtained results will now be preferably focused more on disturbance step responses.

**Figure 6:**
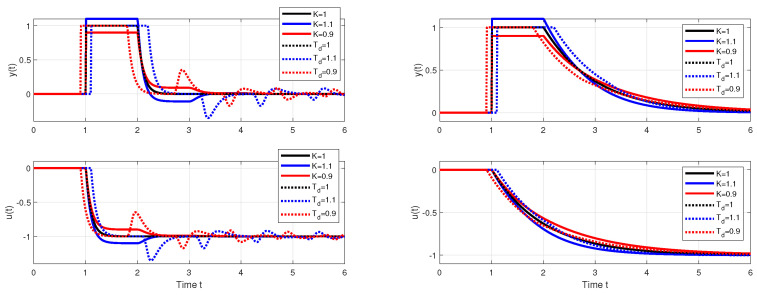
Disturbance responses of PrI controller for nominal and perturbed setting with $\tau_{f}=0.1$ (**left**) and $\tau_{f}=1$ (**right**).

Figure 4 and Figure 6 show that, for $\tau_{f}=0.1$, the step responses are much more sensitive to deviations between *K* and $K_{m}$, or $T_{d}$ and $T_{m}$ than for $\tau_{f}=1$. Thereby, the levels of shape-related deviations in Figure 4, Figure 5 and Figure 6 correspond to the inequalities

(31) $$TV_{0}\left(y\right)\leq \epsilon ;\;TV_{0}\left(u\right)\leq \epsilon$$

visualized for the vector of ϵ values

(32) $$v_{\epsilon }=\left[0.001\;0.002\;0.005\;0.01\;0.02\;0.05\;0.1\;0.2\;0.5\;1\right]$$

by colors ranging from red (with the shape-related deviations below ϵ=0.001) to dark blue. In red areas, setpoint responses can be considered nearly monotonic (MO). For simplicity, disturbance step responses with a monotonic return to zero will also be denoted as monotonic (MO), although in total they consist of two MO intervals forming 1P shapes. IAE values are visualized by contour curves.

The intuitively chosen nominal setting with the values $K_{m}=K$ and $T_{m}=T_{d}$ (marked with a circle) is shown to lie near to the boundary of the MO area mapped with ϵ=0.001. (For ϵ→0, it should lie on the MO border.) This applies both for $\tau_{f}=0.1$, where the width of the MO area is narrower than the width of the quantization step in the evaluation and visualization of the PP, but also for $\tau_{f}=1$, where the position of the optimal nominal setting at the borders of the MO area is much clearer.

**Remark** **3** (The basic problem of the Reswick concept). *Because the used process model (6) is associated with various uncertainties, the achievement of stable transients with satisfactory shapes is conditioned by the possibility of locating the working point specified by $K_{m}$ and $T_{m}$ in the PP area with satisfactory low shape deviations. They are much narrower for small values $\tau_{f}\approx 0$ than for $\tau_{f}\approx 1$ or even $\tau_{f}>1$. The historical failure of Reswick’s method [41] can also be explained by the fact that it was formulated for $\tau_{f}=0$. At the time, however, there were no simple possibilities for analog implementation of dead-time models, but also no simple methods for deeper performance analysis of systems with an explicit dead-time model. And the simple performance analysis methods were also absent when the PPI controller was introduced (25). The possibilities provided in this direction by using the method based on maximum sensitivity functions are far less informative than with PPM.*

The PP calculated for the PrI controller can also be used to visualize the properties of the PPI controller. If applying the obtained information to the setting of PPI controller (25) [47] with the value $T_{1m}\approx T_{1}$, it would be possible to state that the author chose a setting corresponding to a more robust situation with $\tau_{f}=1$. However, this does not necessarily mean that the achieved robustness met the requirements of each practical task, whether it was sufficiently high or too low. This is the principal advantage of PPM, namely that it makes it possible to discuss numerous traditional approaches, which in some contexts may give excellent properties, but can also be not flexible enough to cope with whole variety of possible practical requirements. They can be pre-programmed with a possibly unnecessarily high reserve of the nominal tuning (distance of the corresponding working point from the border of admissible performance) and mostly they do not make it possible to balance dynamics of the considered setpoint and disturbance step responses. In this sense, PPM makes it possible to replace all existing tunings by a single application, while optimally fitting the specified performance requirements without any redundant precaution and safety margin.

## 5. PP Based Controller Design

The controller design could be specified by requirements, for a given process (6), to keep the IAE values (23) as small as possible by choosing appropriate $K_{m},T_{m}$, and the filter equivalent delay $T_{e}$. Thereby, it is important to remember that such an approach will be limited by the following:

- Validity of relation IAE=IE conditioned by not changing the control error sign equivalent to $TV_{0}\left(y_{s}\right)=0$ and $TV_{1}\left(y_{d}\right)=0$;
- Validity of the used process model;
- The limits imposed on the dynamics of the corresponding closed loop.

The first type of limitation requires that the filter time constants $T_{f}$ influencing not only the measurement noise attenuation, but also the closed-loop robustness, should not be arbitrarily low, especially with respect to dead-time uncertainty. This is caused by the fact that several possible loop time constants, included (for the sake of model simplicity) into the model dead-time $T_{d}$, become apparent especially in situations with $K_{m}=K,T_{m}=T_{d}$ lying in PP on the MO border. A more robust setting (paid by longer responses) can be expected when moving the working point deep into the red areas.

Thus, in a robust PrI controller design, the task is to choose parameters $K_{m},T_{m}$, and a low-pass filter $Q_{n}\left(s\right)$ characterized by $T_{e}=nT_{f}$. This is used in PDO and also as a pre-filter $F_{p}\left(s\right)$. The controller setting has to guarantee the fastest possible dynamics of the setpoint and disturbance responses and thereby fulfill the shape-related input and output constraints. At the output, they can be formulated as

(33) $$TV_{0}\left(y_{s}\right)\leq \epsilon_{ys}\;;\;TV_{1}\left(y_{d}\right)\leq \epsilon_{yd}$$

In other words, the setpoint step responses of the output variable should satisfy a tolerable output deviation from monotonicity (MO) and a tolerable output deviation of the disturbance responses from a 1P response.

Similarly, for input transients, it is required to fulfill the shape-related input constraints

(34) $$TV_{0}\left(u_{s}\right)\leq \epsilon_{us}\;;\;TV_{1}\left(u_{d}\right)\leq \epsilon_{ud}$$

with some specified positive $\epsilon_{us}$ and $\epsilon_{ud}$. When wishing to obtain responses with ideal shapes, which play a special role in achieving invariance against process uncertainty [48], it would be required to work with

(35) $$\epsilon =\epsilon_{ys}=\epsilon_{yd}=\epsilon_{us}=\epsilon_{ud}\to 0$$

The always limited precision of control and also that of computer simulations can be respected by some ”sufficiently” small limits chosen with relation to the considered signals of the input and output, e.g., as

(36) $$\epsilon =\epsilon_{ys}=\epsilon_{yd}=\epsilon_{us}=\epsilon_{ud}=0.001$$

PP-based PrI controller design has been firstly treated for setpoint responses with n=1 and $T_{p}=T_{f}$ in [22]. Experience gained in dealing with higher-order DOB-based controllers for the first-order plants gave motivation to come back to PrI controller tuning with a question, namely how do its closed-loop robustness and noise attenuation depend on the order of the low-pass filter $Q_{n}$ (20).

The quasi-continuous controller design has to respect the basic requirement that the sampling period *T* remains sufficiently short with respect to the filter time constant $T_{f}$ used. For the maximal considered order *n* and $\tau_{e}=T_{e}/T_{m}$, it can be expressed by the condition

(37) $$T_{fmin}=T_{emin}/n>>T$$

### 5.1. Robustness Aspects

In practical applications, loop parameters are always known just with some finite precision, i.e., with some degree of uncertainty. Process models can vary in time, due to changes in the operating point (non-linear processes). Their parameters can only be identified with a finite precision. As, e.g., discussed in [47], dead-times typically occur in processes with mass, or information transportation. The dead-time depends on the time necessary to take the material from the position of the supply to the position of the sensor. Thus, it is inversely proportional to the speed of a material, or information flow. If the flow speed varies, so does the dead-time. Similarly, process model parameters are obtained by approximating a more complex non-linear dependence around the selected working point. When it changes; for example, as a result of acting disturbances, the model parameter also changes. Thus, another limitation of the controller design is the consideration of variables, namely uncertain model parameters in (6).

PPM was developed during the research stay of the first author of this contribution at FernUniversität in Hagen in the years 2008–2011. Later, the work carried out in cooperation between FernUni and STU in Bratislava was interrupted by administrative changes concerning both workplaces. Until then, the results concerning robust control systems with uncertain parameters managed to appear in top journals only with regard to the control of processes approximated by the IPDT model $K_{s}e^{-T_{d}s}/s$ using PI controllers [48] and their model-based modification using the disturbance observer (DOB) with inverse process model. The results regarding the robust control of systems approximated by the model (6) were published in a just “preliminary” manner in the workshop materials of the NIL project [22]. To the given topic was also somehow related the work [23]. Before the definitive interruption of this research work, it still managed to be finished as a conference contribution to the robust control of the (6) model [24]. However, within the scope of a standard conference contribution, it was not possible to insert all the important explanatory details. And because during the turbulent development after the end of the initial stage of work on PPM, several records about this task were lost, now, when these results are obviously relevant, for example, for solving MEG problems, there is nothing left but to laboriously obtain them again. In doing so, we will mainly rely on the work [48]. This work with respect to robust PPM-based design of the PrI controller states two important theorems.

The first of them (marked in [48] as Theorem 2) firstly presumes the existence of PP of the 2DOF PI controller with the IPDT model calculated by performance mapping with consideration of model parameters $K_{s}=1$ and $T_{d}=1$. Then, it specifies how it is possible to obtain from this PP actual results enabling optimal and robust setting of the given PI controller for arbitrary non-zero model parameters $K_{s}\neq 0$ and $T_{d}\neq 0$.

The second theorem (marked as Theorem 3) says that the invariance of performance against changes in $T_{d}$ can only be achieved by placing an uncertainty curve segment (UCS) into the area of PP with zero shape-related deviations of the input and output. Thereby, the term UCS was used to denote the set of all possible working points corresponding to an uncertain interval parameter of the dead-time model $T_{d}\in \left[T_{min},T_{max}\right]$.

With regard to the fact that more than ten years have elapsed since the publication of these two theorems, and that they have not been used in the context of controller design for other processes, we assume that they were not formulated in a sufficiently inspiring way. Therefore, in this contribution, we will try to derive them in a slightly different way for two uncertain parameters of the model (6)

(38) $$\begin{array}{c} K\in [K_{min},K_{max}];\\ T_{d}\in \left[T_{min},T_{max}\right]. \end{array}$$

### 5.2. Effect of Gain Uncertainty

As already mentioned, controller design for an uncertain process gain $K\in [K_{min},K_{max}]$ has already been discussed in [24]. The goal was to find a system setting enabling the achievement of minimum summary values $IAE=IAE_{s}+IAE_{d}$ including both the setpoint and the disturbance responses. Now, with regard to the specifics of MEG, it will be more important to focus on disturbance responses by finding settings that minimize the value of $IAE_{d}$ while respecting chosen shape-related constraints for both the setpoint and disturbance steps. When interpreting such a situation geometrically in the PP, its dimension can be decreased to 2D by considering one tuning parameter fixed, e.g., as $T_{m}=T_{d}$. Then, in the PP plain $\left(\kappa ,\tau_{f}\right)$, changes in the process gain *K* with respect to a fixed tuning $K_{m}$ influence possible values of a horizontal coordinate $\kappa =K/K_{m}$. For a chosen value $K_{m}$, the limit dimensionless values will be

(39) $$\kappa_{min}=K_{min}/K_{m}\;;\;\;\kappa_{max}=K_{max}/K_{m}$$

With $K\in [K_{min},K_{max}]$ and a constant $\tau_{f}=T_{f}/T_{m}$, these limit values define an uncertainty line segment (ULS) in the $\left(\kappa ,\tau_{f}\right)$ plane.

Next, inspired by Theorem 2 in [48], we will propose a hypothesis concerning the recalculation of performance measures from PP items generated for a process model with parameters K=1 and $T_{d}=1$. We will also explore the invariance of performance measures against changes in model parameters on regions with zero shape-related deviations. Then, we will verify the hypothesis with analysis of the PP by simulations.

**Remark** **4** (Invariance to changes in model parameters). *By appropriately setting the controller parameters $K_{m},T_{m}$, and $T_{e}$, it is possible to ensure the invariance of only those performance measures that do not directly depend on possibly variable process parameters K and $T_{d}$. For this reason, it is certainly impossible to achieve the invariance of $IAE_{s}$ (23) with respect to changes in K. However, the invariance of $IAE_{d}$ with respect to changes in K is not excluded, nor the invariance of $IAE_{s}$ and $IAE_{d}$ with respect to changes in $T_{d}$. We will also investigate the possibilities of achieving invariance of the shapes of setpoint and disturbance step responses given by zero deviations of the relevant shape-related deviations.*

**Hypothesis** **1** (Performance invariance against uncertainty in *K* over ULS). *For a PrI controller acting on the process with an uncertain gain K (38), (39) with the corresponding ULS located in areas with $TV_{0}\left(u_{s}\right)=0$, $TV_{0}\left(y_{s}\right)=0$, $TV_{1}\left(y_{d}\right)=0$, and $TV_{0}\left(u_{d}\right)=0$, the closed-loop performance expressed in terms of these measures does not depend on the actual value of K.*

When testing the above hypothesis on an illustrative example, consider a 2D PP of PrI controller mapped for K=1, $T_{m}=T_{d}=1$, T=0.01, t∈[0,50], and n=1 over 41 × 41 points with

(40) $$K/K_{m}\in \left[0.2,2.2\right];\;T_{f}/T_{m}\in \left[0.2,2.2\right];\;\Delta \kappa =\Delta \tau_{f}=0.05.$$

**Figure 7:**
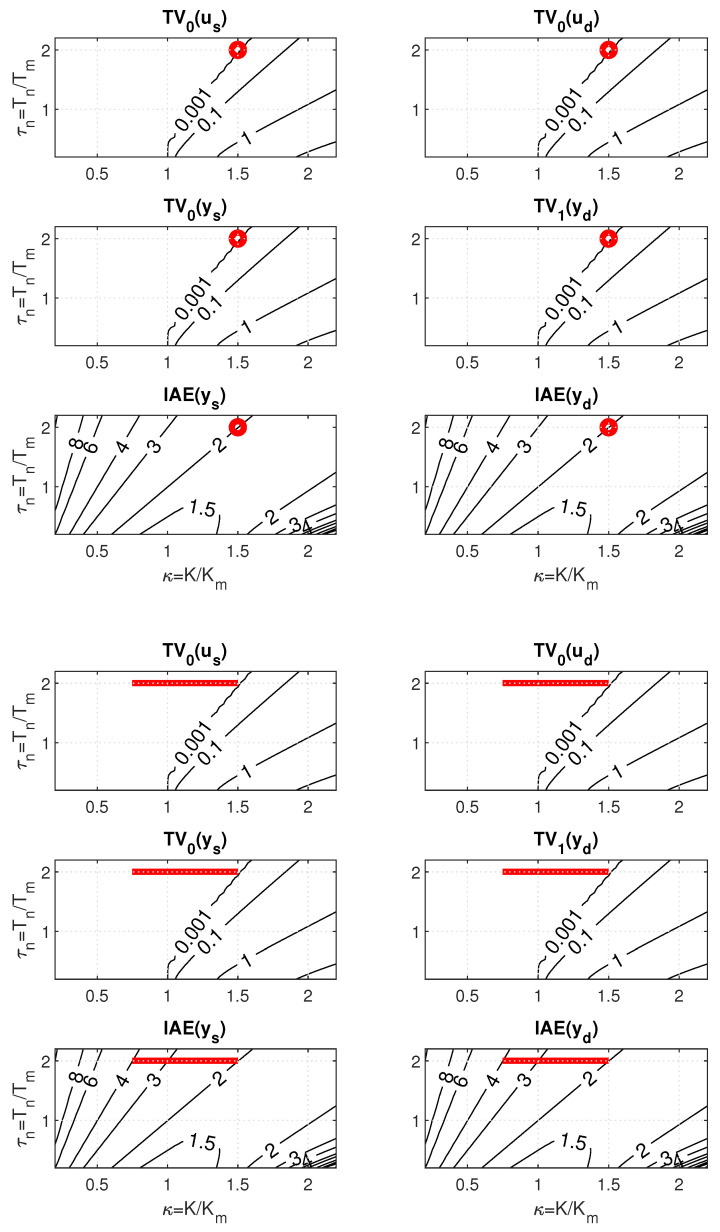
Nominal and robust PrI controller tuning in 2D PP (40) for $K_{max}/K_{min}=1$ (**above**) and $K_{max}/K_{min}=2$ (**below**); $T_{fmin}=2$.

For the minimal admissible value of the filter time constant specified as $T_{fmin}=2$, the sweeping in PP carried out for minimal $IAE_{d}$ with $K_{max}/K_{min}=1$ and the minimal shape-related deviations yields the controller gain $K_{m}\approx 2/3$ with $T_{f}\approx T_{fmin}=2$ (see Figure 7). When the uncertainty specified by $K_{max}/K_{min}$ increases, with the given limitation on $T_{fmin}$, the right corner of the corresponding ULS is located at the point found for the nominal setting. The left limit point thereby moves through the area with minimal shape-related deviations to the left. At the same time, all ULS points correspond to the same $IAE_{d}$ value as the limit nominal setting. To determine a maximal uncertainty increase $K_{max}/K_{min}$ with the value of $IAE_{d}$ invariant to the decrease of $K_{min}$ (39), it would be necessary to expand the scope of the PP mapping.

However, when plotting the ULS in the picture drawn for the PP window in Figure 7 obtained with K=1, the $IAE_{d}$ values along ULS change from $IAE_{d}=2$ to $IAE_{d}=4$. The apparent contradiction will be later explained by a new hypothesis (theorem proposal).

**Figure 8:**
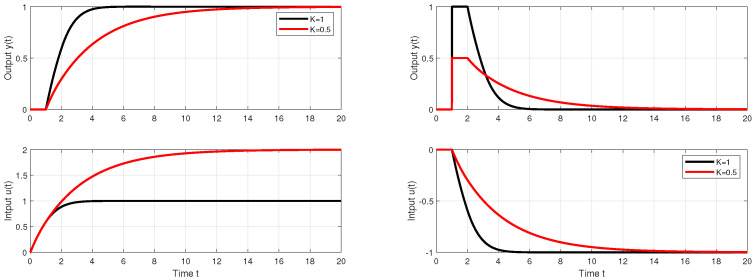
Setpoint and disturbance responses corresponding to PrI controller tuning according to Figure 7 for a gain uncertainty $K_{max}/K_{min}=2$ with invariance of $IAE_{d}$ values.

Before that, however, it is possible to comment the setpoint and disturbance step responses in Figure 8 illustrating the specifics of the robust design used. According to (23), by placing the ULS in areas with almost zero values of the shape-related deviations, it is possible to guarantee for all points of ULS approximately the same values of $IAE_{d}\approx 2$, i.e., in the limit for both $K_{min}$ and $K_{max}$. The value of $K_{m}\approx 2/3$ corresponds to $T_{d}+T_{f}\approx 3$. With respect to the presence of the *K* parameter in the expression for $IAE_{s}$ (23), however, it does not guarantee the invariance of this performance measure with respect to changes in *K*. Robustness also refers to the invariance of shapes of both step responses with respect to changes in *K*.

A more detailed explanation of the apparent paradox, when all points of the considered ULS located in the area with (approximately) zero values of shape deviations guarantee the same value of $IAE_{d}$, but the values of this parameter change in the corresponding window Figure 7 right, is provided by the following theorem. It is similar to Theorem 2 in [48].

**Theorem** **2.** *Let us consider PP including items $\bar{IAE}\left({\bar{y}}_{s}\right)$, $\bar{IAE}\left({\bar{y}}_{d}\right)$, ${\bar{TV}}_{0}\left({\bar{y}}_{s}\right)$, ${\bar{TV}}_{1}\left({\bar{y}}_{d}\right)$, ${\bar{TV}}_{0}\left({\bar{u}}_{s}\right)$, and ${\bar{TV}}_{0}\left({\bar{u}}_{d}\right)$ generated for process with $K=1,T_{d}=1$ over chosen grid of points $K_{m},T_{m},T_{f},n$ by simulating setpoint responses ($w=1,d_{i}=0$) with output and input ${\bar{y}}_{s}\left(\tau \right)$, ${\bar{u}}_{s}\left(\tau \right)$ and input disturbance responses ($w=0,d_{i}=1$) with ${\bar{y}}_{d}\left(\tau \right),{\bar{u}}_{d}\left(\tau \right)$. Performance measures of these responses are stored and expressed over grid of normalized variables*

(41) $$\begin{array}{c} \kappa =K/K_{m}\in \left[\kappa_{min},\kappa_{max}\right];\;\tau_{d}=T_{d}/T_{m}\in \left[\tau_{dmin},\tau_{dmax}\right]\;;\;\;\;\tau_{f}=T_{f}/T_{m}\in \left[\tau_{fmin},\tau_{fmax}\right] \end{array}$$

*Then, the PP items $IAE\left(y_{s}\right),IAE\left(y_{d}\right)$ corresponding to any process parameters $K,T_{d}$ and setting $K_{m},T_{m},T_{f},n$ belonging to the range (41) with the corresponding responses $y_{s}\left(t\right),u_{s}\left(t\right)$, $y_{d}\left(t\right)$, and $u_{d}\left(t\right)$ may be calculated according to*

(42) $$\begin{array}{c} IAE\left(y_{s}\right)=T_{d}\;\bar{IAE}\left({\bar{y}}_{s}\right)\;;\;\;IAE\left(y_{d}\right)=KT_{d}\;\bar{IAE}\left({\bar{y}}_{d}\right) \end{array}$$

The linear decrease in $IAE_{d}$ values with the decrease in $K/K_{m}$ values along the ULS seems to compensate for the linear increase in ${\bar{IAE}}_{d}$ along the ULS, thus ensuring the invariance of this performance measure with changes in *K*. We will further verify the same effect for the case of uncertainty regarding $T_{d}$.

### 5.3. Effect of Dead-Time Uncertainty

Unlike the invariance in IAE values against *K* changes, which cannot be achieved in the case of $IAE_{s}$ for setpoint step responses due to appearance of *K* in the corresponding expression for $IAE_{s}$ in (23), the situation changes when considering perturbations in $T_{d}$. The actual loop delay $T_{d}$ does not appear in any of the relations (23).

Consider a 2D PP of PrI controller mapped for $K=K_{m}=1$, $T_{d}=1$, T=0.01, t∈[0,120], and n=1 over 41 times 41 points with

(43) $$T_{d}/T_{m}\in \left[0.2,2.2\right];\;T_{f}/T_{m}\in \left[0.2,2.2\right];\;\Delta \tau_{d}=\Delta \tau_{f}=0.05.$$

**Figure 9:**
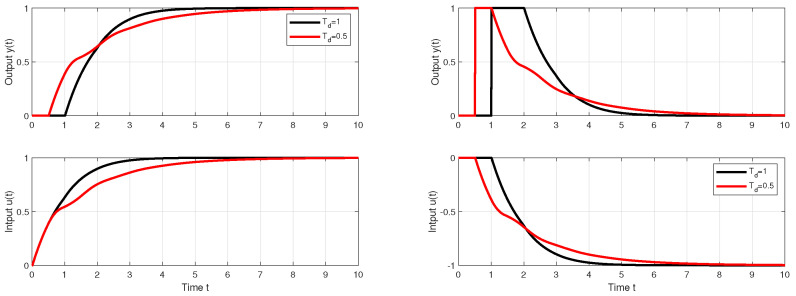
Limit setpoint and disturbance responses corresponding to PrI controller tuning according to Figure 10 with dead-time uncertainty $T_{max}/T_{min}=2$ and invariance in both the $IAE_{s}$ and the $IAE_{d}$ values.

**Figure 10:**
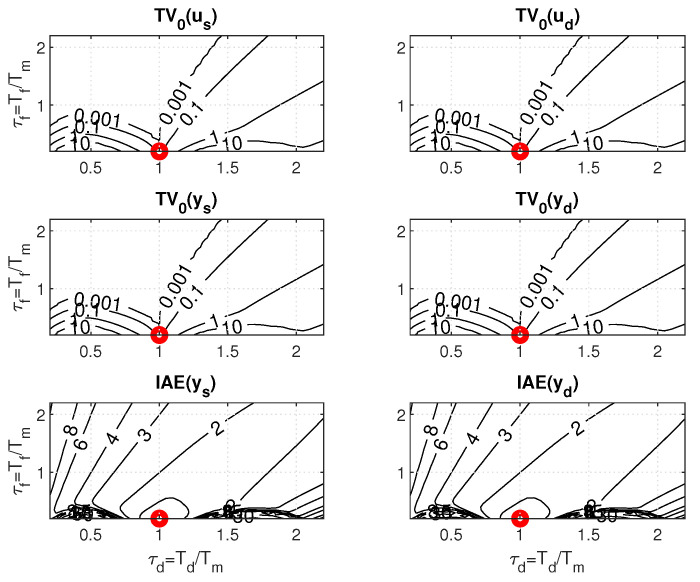

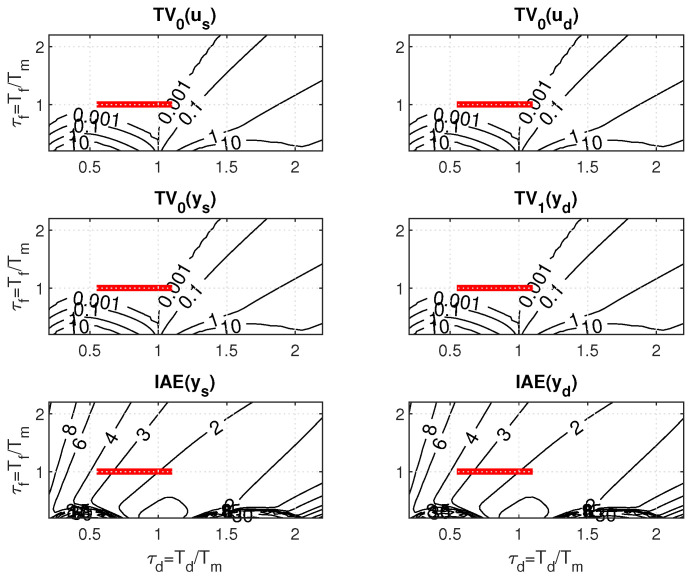
Nominal and robust PrI controller tuning in 2D PP (40) $T_{max}/T_{min}=1$ (**above**) and $T_{max}/T_{min}=2$ (**below**).

The setpoint and disturbance step responses in Figure 9 illustrate the specifics of the robust design used with respect to dead-time uncertainty. Since the IAE values (23) do not explicitly depend on $T_{d}$, by placing the ULS corresponding to $T_{d}$ perturbations into an area with almost zero values of shape deviations (see Figure 10), it guarantees for all points of ULS approximately the same values of $IAE_{d}\approx 2$, i.e., both for $T_{d}=1$ and $T_{d}=2$. The value of $T_{m}\approx 2/3$, corresponds to $T_{d}+T_{f}\approx 3$. Robustness also refers to the invariance in shapes of the responses with respect to changes in $T_{d}$.

The increase in ${\bar{IAE}}_{s}$ and ${\bar{IAE}}_{d}$ values when decreasing $T_{d}$ along the ULSs from the value of ≈2 to the value of ≈4 is compensated by the twofold decrease in $\tau_{d}=T_{d}/T_{m}$ evident from the relations (42), which guarantee the invariance in both $IAE_{s}$ ad $IAE_{d}$.

### 5.4. Use of Higher-Order Low-Pass Filters

The motive for using PrI controllers with consideration of higher-order low-pass filters (20) can be the achievement of better measurement noise attenuation, but also active compensation of the additional time constant(s) of the sensor (feedback) dynamics according to Figure 3. At the same time, however, it will be necessary to examine how the use of higher-order filters affects the robustness of the system. Because with an uncertain gain *K* everything depends on the limitation (37), we will further focus on the uncertainty in $T_{d}$, where changes in PP play the main role.

The procedure for designing PrI controllers using PPM with a filter degree n>1 is basically the same as for n=1. Thus, to establish a comparative framework, for n∈[2,3] PPs will be specified according to

(44) $$T_{d}/T_{m}\in \left[0.2,2.2\right];\;T_{e}/T_{m}\in \left[0.2,2.2\right];\;\Delta \tau_{d}=\Delta \tau_{e}=0.05;\;T_{f}=T_{e}/n;\;\tau_{f}=\tau_{e}/n.$$

**Figure 11:**
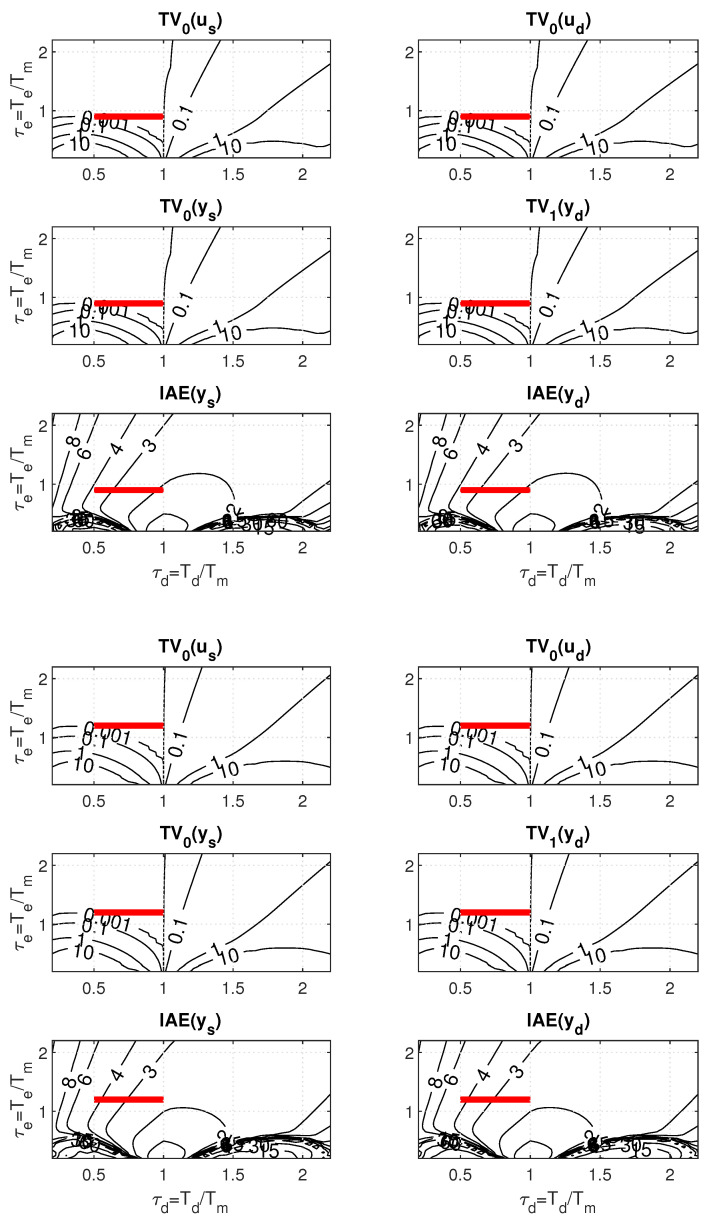
Robust PrI controller tuning in 6 windows of 2D PP with dead-time uncertainty $T_{max}/T_{min}=2$ for n=2 (**above**) and n=3 (**below**).

Thereby, it was only necessary to replace $T_{f}$ with $T_{e}$ in (43) and add $T_{f}=T_{e}/n$. The same treatment also applies to the dimensionless parameters $\tau_{f}$ and $\tau_{e}$. Changes in the six windows of PP caused by an increase in *n* are manifested firstly by a change in the areas with approximately zero shape-related deviations constrained by the contours corresponding to ϵ=0.001 (compare Figure 11 with Figure 10). Subsequently, of course, the contours corresponding to the IAE values also change.

**Figure 12:**
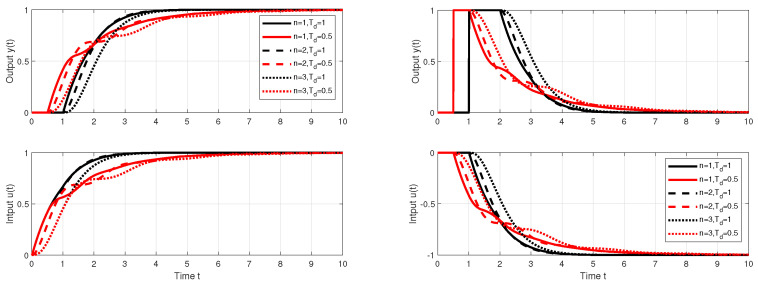
Limit setpoint and disturbance responses corresponding to PrI controller with filter order n∈[1,3] and robust tuning according to Figure 10 and Figure 11 for $T_{max}/T_{min}=2$.

For the case of the dead-time uncertainty, the above described PP-based robust PrI controller design leads for n∈[1,3] to a gradual increase in both $IAE_{s}$ and $IAE_{d}$ values (see Table 1). For n=2, it is an increase of about 4%, and for n=3 of about 20% in comparison to n=1. The corresponding step responses are smoother, but slightly more wavy (Figure 12).

However, before formulating conclusions about robustness decrease with increasing *n*, the evaluation of performance measures of the circuit with the addition of non-zero measurement noise must also be taken into account. In Matlab/Simulink, such a noise was generated by the Uniform Random Number block with sampling period T=0.01 and amplitude $\delta_{n}<0.1$. Achieved performance measures are shown in Table 2.

It is worth noting that, for n=1 and $T_{d}=1/2$, the values of $IAE_{s}$ decrease under measurement noise by about 2% with respect to $T_{d}=1$. In the case of $IAE_{d}$ values, the given decrease is smaller (below 1%). In the circuit without measurement noise, the fluctuations of both IAE values were significantly lower (see Table 1). So, although the dependence of shape deviations and the combined cost function on changes in *K* relatively increases, their absolute deviations decrease significantly as *n* increases.

It is worth noting that, for n=1 and $T_{d}=1/2$, the values of $IAE_{s}$ decrease by about 2%. In the case of $IAE_{d}$ values, the given decrease is smaller (below 1%). In the circuit without measurement noise, the fluctuations of both IAE values were significantly lower. Significantly higher percentage changes after a change in $T_{d}$ occur in the shape-related performance measures of the input and output of the process. However, the absolute values of these deviations decrease significantly with increasing *n*. Increasing the range of the low-pass filter *n* is clearly shown to be a benefit from the point of view of using the combined cost function *J* related to the setpoint, or disturbance responses ($J_{s}$ and $J_{d}$).

## 6. Discussion

Approximation of the process by gain+dead-time represents the simplest linear model of stable systems. Therefore, when examining the historical development of automatic control, it is possible to find several solutions based on this model. When discussing them with focus on analog solutions, early discrete-time controllers implemented by electro-mechanical devices have been omitted [40]. A continuously working I-controller was designed to control such a model already in the automatic control textbook from the early period of its development [40]. It was a typical representative of the solution from DC0 trying to achieve as fast as possible monotonic responses on the output with monotonic input signals. By simply expanding the simplest process model by another time constant and its cancelling by inverse term, a PI controller would be created. Such a design would keep its dynamics in DC0.

In roughly the same time period, however, the three-term Taylor Fulscope Controller, labelled later with the abbreviation PID, was created, which improved the dynamics in connection with achieving control signals consisting of more than one monotonic interval. This led to the dominant use of PI and PID controllers in industrial practice. But they were primary solutions from DC1. Since the consideration of dynamic classes of control did not appear in the established theory of PID control, no one addressed the emerging problems. However, without a clear distinction between solutions from different dynamic classes, attempts to systematically design entire families of controllers, such as the recently published work [49], will hardly be successful. Furthermore, it was only a matter of time when the ever-increasing speed of achieved transients in PID control would manifest itself in the need to rediscover already known conclusions from the period of development of minimum-time control.

The disturbance–response–feedback proposed by Reswick [41] came at a time when the implementation of dead-time models with the analog technique was too difficult. Even if it was noticed later by the first textbooks of discrete-time control based on digital computers (e.g., [43,44]), it was already too late to promote against the established practices of the industry. Hägglund [46,47] tried much more to respect these habits of practice with the presentation of the PPI controller as a special case of PID control. But even his approach did not enter the consciousness of engineering practice in such a way that it was noticed by new works in the field of MEG. Even though, in the meantime, the use of PPM [22,23,24] made it possible to design the required controllers with far more accurate reasoning of the uncertainty of the relevant system model.

The short analysis in this paper showed that circuits with inversion of the dead-time term approximation have problems with the oscillatory disturbance response when the implementation filter time constant is reduced. Circuits with predictive disturbance observer are facing the only limitation that the time required for their implementation, affecting the choice of the sampling period *T*, should be sufficiently small compared to the required $T_{f}$ value. The choice of this time constant dominantly affects the speed of disturbance responses (see $IAE_{d}$ values (22), (22)), robustness of the solution, and measurement noise attenuation.

The use of ADRC in connection with MEG can be considered more a consequence of fashion trends than an effective reasoning of the dynamics of controlled processes. ADRC was principally developed on the basis of state-space approaches to disturbance observer control formulated for the use of integral models. It also enables the simple consideration of dead-time in the DOB design [21], but the overall design of the controller is unnecessarily complicated compared to the design of the PrI controller. A detailed comparison of both approaches deserves a separate publication in the future.

From the summary of robust settings for all considered situations with dead-time uncertainty

(45) $$\begin{array}{c} n=1;\;K_{m}=1;\;T_{m}=0.9091;\;T_{e}=T_{f}=0.9091;\\ n=2;\;K_{m}=1;\;T_{m}=1;\;T_{e}=0.9000;\;T_{f}=0.45;\\ n=3;\;K_{m}=1;\;T_{m}=1;\;T_{e}=1.2000;\;T_{f}=0.4; \end{array}$$

it turns out that, when *n* increases, there is no simple trend that would allow for precise approximation based on simple formulas. It would perhaps be possible to find out more by applying calculations with significantly finer level-quantization of adjusted parameters.

Rather, it will be interesting to use the wide possibilities of parallel processing in connection with cloud computing. In this context, it would be simple to take into account the two uncertain parameters already considered in the work [22], supplemented by another uncertain parameter approximating the time constant of the used sensor.

This shows the development of the issue in the historical context, the benefits of solving it using the performance portrait method, and the possibility of application to control magnetoencephalography processes using optically pumped magnetometers.

It is also interesting that, in more than ten years since the publication of the first results, there has not yet been a wider use of the performance portrait method. The number of reactions to the PPM is not low, but we are still aware of a separate repetition of the entire proposal or its expansion. If we were to take a closer look at the cited publications, the work [50] clearly characterizes them. Its authors rejected the PPM presented in [48] as a brute force approach and instead, for the system $K_{s}e^{-T_{d}s}/s$ with interval parametric uncertainty, they proposed an alternative based on the minimization of IE values (maximization of the integral amplification $K_{i}$) subject to robustness constraints with the use of maximum sensitivity functions [51,52,53]. The problem of the uncertainty of process parameters is solved by considering two IPDT models and, as they say, with a minimal degree of conservatism. It calculates and approximates the polygon of feasible controller parameters using five straight line segments. A direct application of tedious calculations results in the tuning rule depending on the specifications of sensitivity constraints $M_{s}$ and $M_{t}$, and also on the value of $K_{smax}/K_{smin}$. When comparing, it should be noted that [48] deals with the design of a 2DoF PI controller, while [50] deals with a 1DoF PI controller. Note that overshoot and settling time for step setpoint response and maximum deviation and settling time with respect to the standard 5% criterion for step disturbance response were considered. The overshoot for setpoint response is always less than 30%. They complain that [48] did not use the same performance measures. At the same time, however, they overlook that their choice of performance measures is contained in the used deviations from ideal shapes. However, the PPM itself makes it possible to arbitrarily add other performance measures to the PP and design transient responses with arbitrary deviations from ideal shapes. That is one of its strengths. However, one of the basic goals of [48] was to achieve invariance with respect to $T_{d}$ changes. In doing so, it was shown that invariance can only be achieved with near-ideal responses. And the procedures from the work [50] do not allow that. Moreover, the overshoot for setpoint response less than 30% is simply unacceptable for many applications. So the choice of new procedures and performance measures is not only a matter of taste and respect for tradition. If the output of the [50] work should be modified in any way, it will be necessary to repeat all the “tedious calculations” used. And repeatedly searching for the optimal parameters of PI and PID controllers with variously changed search contexts has really become a fashionable thing and unnecessarily distracts the readers’ attention. While with PPM, it is enough to change the performance specifications appropriately for the desired new solution. It is therefore time to reevaluate the definition of “brute force approach” in relation to the optimal design of automatic controllers.

Apparently, however, the proposal of the PPM itself is more complicated than it might seem from its simple principle of “verifying all relevant solutions”. In the future, it will be necessary to do significantly more in terms of sharing the available solutions with a wider community of users. After acceptance of this contribution, therefore, all used programs will be made available to the public.

## 7. Conclusions

This paper provides a brief overview of recent works in the field of magnetoencephalography (MEG) and shows that, from the point of view of automatic control, these are tasks with dominant process dynamics concentrated on feedback. From the works that analyze this dynamic in more detail, it appears that these are tasks with a dominant dead-time supplemented by the time constant of the sensor. In the next part of the contribution, this paper gives a short overview of the work on dominant dead-time systems, the calculation of which begins with the design of integrating (I) controllers from the period of the Second World War. Among the later approaches, it recalls the oldest known method for active dead-time compensation in control systems introduced by Reswick as disturbance–response feedback and later Hägglund’s predictive proportional–integrative (PPI) controller.

The work then develops model-based controller design for processes with dominant dynamics of the used sensors approximated by the simplest gain + dead-time model. It shows the limited possibilities of speeding up processes offered by the simplest I-controllers. The use of PI controllers or also of PID or PIDA controllers is interpreted as an approximation of the inversion of the dead-time element according to Taylor series expansion with the consideration of an increasing number of replacement terms. However, even increasing the degree of approximation does not lead to results comparable to the compensation of disturbances reconstructed by a simple predictive disturbance observer resulting from disturbance–response feedback. The combination of disturbance compensation with a simple static setpoint feed-forward supplemented with a pre-filter leads here to a solution called a predictive I-controller (PrI).

Based on the performance measures needed to evaluate the ideal input and output responses, and deviations from such responses, this paper then shows the performance portrait (PP) of the system with a predictive I-controller proposed for the simplest gain+dead-time model calculated by mapping the closed-loop properties over a map of dimensionless loop parameters. This PP explains what were the roots of the failure of Reswick’s disturbance–response feedback. When extending the model with a sensor time constant, it also indicates a possible interpretation of performance portrait corresponding to Hägglund’s PPI controller. Next, this paper showed how to design PrI controller parameters using PPM so that the achieved transient responses are as fast as possible, but at the same time invariant to the indeterminate parameters of the process specified by interval model parameters. Further work planned in this area will deal with a detailed comparison of existing solutions from the area of MEG based on ADRC with PrI, and specifically with predictive PrPI controllers. 

## Figures and Tables

**Table 1:** Use of the filter order on the IAE values, n∈[1,3], no noise.

	${\mathrm{IAE}}_{s}$			${\mathrm{IAE}}_{d}$		
-	n=1	n=2	n=3	n=1	n=2	n=3
$T_{d}=1$	1.8239	1.9050	2.2050	1.8218	1.9030	2.2030
$T_{d}=1/2$	1.8203	1.9017	2.1984	1.8171	1.8984	2.1943

**Table 2:** Use of the filter order on the performance measures of the robustly tuned PrI controller, n∈[1,3], random measurement noise δ<0.1.

	${\mathrm{IAE}}_{s}$			${\mathrm{IAE}}_{d}$		
-	n=1	n=2	n=3	n=1	n=2	n=3
$T_{d}=1$	1.8480	1.9275	2.2258	1.8332	1.9142	2.2130
$T_{d}=1/2$	1.8133	1.8949	2.1918	1.8253	1.9055	2.2009
	$TV_{0}\left(u_{s}\right)$			$TV_{0}\left(u_{d}\right)$		
-	n=1	n=2	n=3	n=1	n=2	n=3
$T_{d}=1$	0.3675	0.0452	0.0209	0.3646	0.0298	0.0100
$T_{d}=1/2$	0.2776	0.0247	0.0021	0.2662	0.0172	0.0057
	$TV_{0}\left(y_{s}\right)$			$TV_{1}\left(y_{d}\right)$		
-	n=1	n=2	n=3	n=1	n=2	n=3
$T_{d}=1$	0.3097	0.0340	0.0186	0.3099	0.0176	0.0018
$T_{d}=1/2$	0.2401	0.0125	0.0006	0.2414	0.0147	0.0025
	$J_{s}$			$J_{d}$		
-	n=1	n=2	n=3	n=1	n=2	n=3
$T_{d}=1$	0.6791	0.0871	0.0466	0.6684	0.0571	0.0220
$T_{d}=1/2$	0.5034	0.0469	0.0046	0.4858	0.0328	0.0125

## Data Availability

Programs for generating data visualized by Figure 4, Figure 5, Figure 6, Figure 7, Figure 8, Figure 9, Figure 10, Figure 11 and Figure 12 will be available after the paper acceptance.

## Abbreviations

The following abbreviations are used in this manuscript:

1P	One-Pulse, response with 2 monotonic segments (1 extreme point)
2D	Two-Dimensional
3D	Three-Dimensional
1DoF	One Degree of Freedom
2DoF	Two Degrees of Freedom
ADRC	Active Disturbance Rejection Control
DC0	Dynamic Class 0
DC1	Dynamic Class 1
DOB	Disturbance Observer
DRDP	Double Real Dominant Pole
FOTD	First-Order Time Delayed
GADRC	Generalized Active Disturbance Rejection Control
I	Integral, Integrating
IAE	Integral of Absolute Error
IPDT	Integrator Plus Dead-Time
MEG	Magnetoencephalography
MO	Monotonic, Monotonicity
MRDP	Multiple Real Dominant Pole
OPM	Optically Pumped Magnetometers
PDO	Predictive Disturbance Observer
PI	Proportional–Integral
PID	Proportional–Integral–Derivative
PIDA	Proportional–Integral–Derivative–Acceleration
PPI	Predictive Proportional–Integral
PP	Performance Portrait
PPM	Performance Portrait Method
PrI	Predictive Integrating Controller
PrPI	Predictive Proportional–Integrating Controller
TV	Total Variation
TV_0_	Deviation from Monotonicity
TV_1_	Deviation from 1P Shape
